# Crystal structure of tris­(hydroxyl­ammonium) orthophosphate

**DOI:** 10.1107/S2056989015018642

**Published:** 2015-10-10

**Authors:** Malte Leinemann, Inke Jess, Jan Boeckmann, Christian Näther

**Affiliations:** a Dräger Safety AG & Co. KGaA, Revalstrasse 1, 23560 Lübeck, Germany; b Institut für Anorganische Chemie, Christian-Albrechts-Universität Kiel, Max-Eyth-Str. 2, 24118 Kiel, Germany

**Keywords:** crystal structure, hydroxyl­ammonium salt, hydrogen bonding

## Abstract

The crystal structure of the title salt, ([H_3_NOH]^+^)_3_·[PO_4_]^3−^, consists of discrete hydroxyl­ammonium cations and ortho­phos­phate anions. The atoms of the cation occupy general positions, whereas the anion is located on a threefold rotation axis that runs through the phospho­rus atom and one of the phosphate O atoms. In the crystal structure, cations and anions are linked by inter­molecular O—H⋯O and N—H⋯O hydrogen bonds into a three-dimensional network. Altogether, one very strong O—H⋯O, two N—H⋯O hydrogen bonds of medium strength and two weaker bifurcated N—H⋯O inter­actions are observed.

## Related literature   

The structure determination of the title compound was undertaken as a part of a project on the synthesis and structural characterization of hydroxyl­ammonium salts with simple inorganic anions. For crystal structures of other hydroxyl­ammonium salts with perchlorate, chloride or sulfate anions, see: Dickens (1969 ▸); Jerslev (1948 ▸); Shi *et al.* (1987 ▸); Mirceva & Golic (1995 ▸).
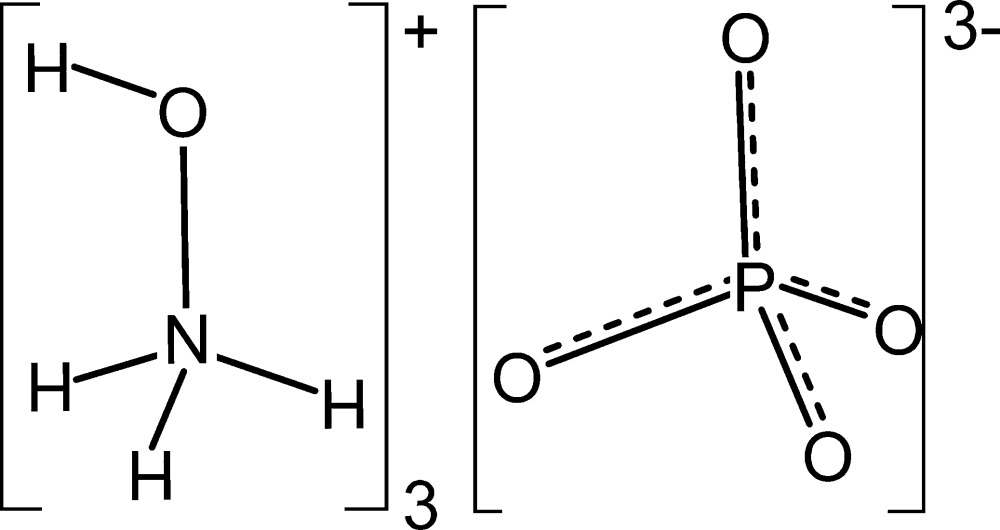


## Experimental   

### Crystal data  

3H_4_NO^+^·PO_4_^3−^
*M* *_r_* = 197.10
Trigonal, 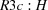

*a* = 10.7072 (9) Å
*c* = 11.0283 (13) Å
*V* = 1094.9 (2) Å^3^
*Z* = 6
Mo *K*α radiation
μ = 0.39 mm^−1^
*T* = 170 K
0.15 × 0.12 × 0.11 mm

### Data collection  

Stoe IPDS-2 diffractometer
5420 measured reflections
647 independent reflections
621 reflections with *I* > 2σ(*I*)
*R*_int_ = 0.047

### Refinement  

*R*[*F* ^2^ > 2σ(*F* ^2^)] = 0.034
*wR*(*F* ^2^) = 0.090
*S* = 1.09
647 reflections
36 parameters
1 restraint
H-atom parameters constrained
Δρ_max_ = 0.24 e Å^−3^
Δρ_min_ = −0.32 e Å^−3^
Absolute structure: Flack *x* determined using 287 quotients [(*I* ^+^)−(*I* ^−^)]/[(*I* ^+^)+(*I* ^−^)] (Parsons *et al.*, 2013 ▸)
Absolute structure parameter: −0.06 (9)

### 

Data collection: *X-AREA* (Stoe, 2008 ▸); cell refinement: *X-AREA*; data reduction: *X-AREA*; program(s) used to solve structure: *SHELXS97* (Sheldrick, 2008 ▸); program(s) used to refine structure: *SHELXL2014* (Sheldrick, 2015 ▸); molecular graphics: *XP* in *SHELXTL* (Sheldrick, 2008 ▸) and *DIAMOND* (Brandenburg, 1999 ▸); software used to prepare material for publication: *publCIF* (Westrip, 2010 ▸).

## Supplementary Material

Crystal structure: contains datablock(s) I, global. DOI: 10.1107/S2056989015018642/wm5223sup1.cif

Structure factors: contains datablock(s) I. DOI: 10.1107/S2056989015018642/wm5223Isup2.hkl

**Fig. 1.**
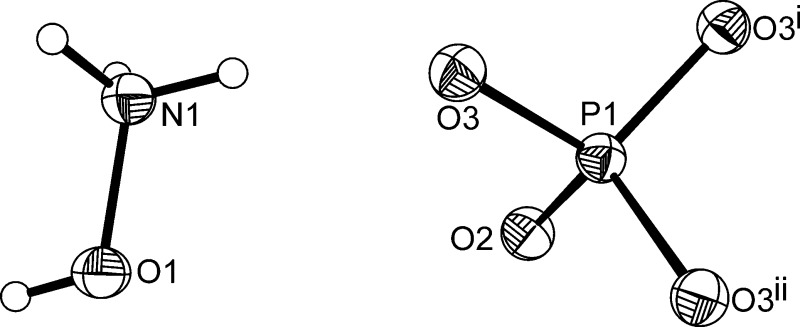
View of the molecular components of the title compound with labelling and displacement ellipsoids drawn at the 50% probability level. [Symmetry codes: i) = -x+y, -x+1, z, ii) = -y+1, x-y, z.]

**Fig. 2.**
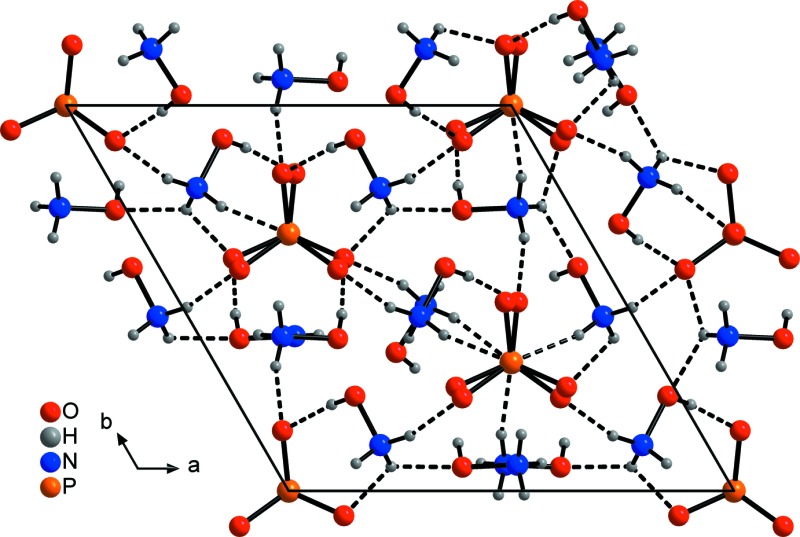
Crystal structure of the title compound in a view along the crystallographic c axis. Intermolecular hydrogen bonding is shown as dashed lines. For clarity, only parts of the hydrogen bonding interactions are shown.

Supporting information file. DOI: 10.1107/S2056989015018642/wm5223Isup3.cml

x y x z; y x y z . DOI: 10.1107/S2056989015018642/wm5223fig1.tif
View of the mol­ecular components of the title compound with labelling and displacement ellipsoids drawn at the 50% probability level. [Symmetry codes: (i) −*x* + *y*, −*x* + 1, *z;* (ii) −*y* + 1, *x* − *y*, *z*.]

c . DOI: 10.1107/S2056989015018642/wm5223fig2.tif
Crystal structure of the title compound in a view along the crystallographic *c* axis. Inter­molecular hydrogen bonding is shown as dashed lines. For clarity, only parts of the hydrogen-bonding inter­actions are shown.

CCDC reference: 1429513

Additional supporting information:  crystallographic information; 3D view; checkCIF report

## Figures and Tables

**Table 1:** Hydrogen-bond geometry (, )

*D*H*A*	*D*H	H*A*	*D* *A*	*D*H*A*
O1H1*O*1O3^i^	0.84	1.70	2.540(3)	172
N1H1*N*1O2^ii^	0.91	1.90	2.785(3)	163
N1H2*N*1O1^iii^	0.91	2.37	3.110(4)	138
N1H2*N*1O3^iv^	0.91	2.20	2.884(3)	132
N1H3*N*1O3	0.91	1.84	2.732(3)	164

Symmetry codes: (i) 
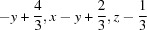
; (ii) 
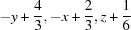
; (iii) 
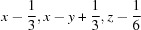
; (iv) 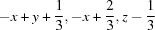.

## supplementary crystallographic information

### S1. Synthesis and crystallization

The title compound tris­(hydroxyl­ammonium) orthophosphate was synthesized by the reaction of 29.4 g H_3_PO_4_ (0.30 mol) with 29.7 g NH_2_OH (0.90 mol) in aqueous solution under cooling. The resulting precipitate was filtered off, washed with mother liquor and dried in vacuum at 343 K for one day. The purity was checked by X-ray powder diffraction. Single crystals suitable for X-ray diffraction analysis were obtained by dissolving 0.5 g of the polycrystalline powder of tris­(hydroxyl­ammonium)­phosphate in 5 ml of water in a snap cap vial, allowing the solvent to evaporate slowly. After a few days colorless block-shaped crystals of the title compound were obtained.

### S2. Refinement

The N–H and O–H hydrogen atoms were located in a difference map but in the final refinement they were positioned with idealized geometry allowed to rotate but not to tip. H atoms were refined with *U*_iso_(H) = 1.5*U*_eq_(N,O) using a riding model with O—H = 0.82 Å and N—H = 0.99 Å, respectively.

### Crystal data

**Table:**

3H_4_NO^+^·PO_4_^3^^−^	*D*_x_ = 1.793 Mg m^−^^3^
*M**_r_* = 197.10	Mo *K*α radiation, λ = 0.71073 Å
Trigonal, *R*3*c*:*H*	Cell parameters from 5420 reflections
*a* = 10.7072 (9) Å	θ = 7.6–59.2°
*c* = 11.0283 (13) Å	µ = 0.39 mm^−^^1^
*V* = 1094.9 (2) Å^3^	*T* = 170 K
*Z* = 6	Block, colorless
*F*(000) = 624	0.15 × 0.12 × 0.11 mm

### Data collection

**Table:**

Stoe IPDS-2 diffractometer	*R*_int_ = 0.047
ω scans	θ_max_ = 29.0°, θ_min_ = 3.8°
5420 measured reflections	*h* = −14→14
647 independent reflections	*k* = −14→14
621 reflections with *I* > 2σ(*I*)	*l* = −15→15

### Refinement

**Table:**

Refinement on *F*^2^	Hydrogen site location: inferred from neighbouring sites
Least-squares matrix: full	H-atom parameters constrained
*R*[*F*^2^ > 2σ(*F*^2^)] = 0.034	*w* = 1/[σ^2^(*F*_o_^2^) + (0.0636*P*)^2^ + 0.3897*P*] where *P* = (*F*_o_^2^ + 2*F*_c_^2^)/3
*wR*(*F*^2^) = 0.090	(Δ/σ)_max_ < 0.001
*S* = 1.09	Δρ_max_ = 0.24 e Å^−^^3^
647 reflections	Δρ_min_ = −0.32 e Å^−^^3^
36 parameters	Absolute structure: Flack x determined using 287 quotients [(I+)-(I-)]/[(I+)+(I-)] (Parsons *et al*., 2013)
1 restraint	Absolute structure parameter: −0.06 (9)

### Special details

**Table:**

Geometry. All esds (except the esd in the dihedral angle between two l.s. planes) are estimated using the full covariance matrix. The cell esds are taken into account individually in the estimation of esds in distances, angles and torsion angles; correlations between esds in cell parameters are only used when they are defined by crystal symmetry. An approximate (isotropic) treatment of cell esds is used for estimating esds involving l.s. planes.

### Fractional atomic coordinates and isotropic or equivalent isotropic displacement parameters (Å^2^)

**Table:**

	*x*	*y*	*z*	*U*_iso_*/*U*_eq_	
O1	0.6408 (2)	0.5792 (2)	0.5089 (3)	0.0230 (5)	
H1O1	0.6827	0.5567	0.4562	0.035*	
N1	0.5202 (3)	0.4522 (2)	0.5550 (2)	0.0203 (5)	
H1N1	0.5515	0.4032	0.6016	0.030*	
H2N1	0.4668	0.3955	0.4922	0.030*	
H3N1	0.4649	0.4767	0.6008	0.030*	
P1	0.3333	0.6667	0.64509 (11)	0.0166 (3)	
O2	0.3333	0.6667	0.5059 (3)	0.0223 (8)	
O3	0.3977 (2)	0.5747 (2)	0.69256 (18)	0.0202 (5)	

### Atomic displacement parameters (Å^2^)

**Table:**

	*U*^11^	*U*^22^	*U*^33^	*U*^12^	*U*^13^	*U*^23^
O1	0.0240 (11)	0.0183 (9)	0.0246 (9)	0.0089 (8)	0.0057 (9)	0.0017 (8)
N1	0.0201 (11)	0.0179 (12)	0.0219 (12)	0.0087 (10)	0.0025 (10)	0.0006 (10)
P1	0.0171 (3)	0.0171 (3)	0.0157 (5)	0.00855 (17)	0.000	0.000
O2	0.0233 (11)	0.0233 (11)	0.0205 (18)	0.0116 (6)	0.000	0.000
O3	0.0207 (10)	0.0200 (9)	0.0200 (10)	0.0103 (8)	−0.0003 (9)	−0.0002 (8)

### Geometric parameters (Å, º)

**Table:**

O1—N1	1.421 (3)	P1—O2	1.535 (4)
O1—H1O1	0.8400	P1—O3^i^	1.548 (2)
N1—H1N1	0.9100	P1—O3^ii^	1.548 (2)
N1—H2N1	0.9100	P1—O3	1.548 (2)
N1—H3N1	0.9100		
			
N1—O1—H1O1	109.5	O2—P1—O3^i^	109.77 (9)
O1—N1—H1N1	109.5	O2—P1—O3^ii^	109.77 (9)
O1—N1—H2N1	109.5	O3^i^—P1—O3^ii^	109.17 (9)
H1N1—N1—H2N1	109.5	O2—P1—O3	109.77 (9)
O1—N1—H3N1	109.5	O3^i^—P1—O3	109.17 (9)
H1N1—N1—H3N1	109.5	O3^ii^—P1—O3	109.17 (9)
H2N1—N1—H3N1	109.5		

Symmetry codes: (i) −*y*+1, *x*−*y*+1, *z*; (ii) −*x*+*y*, −*x*+1, *z*.

### Hydrogen-bond geometry (Å, º)

**Table:**

*D*—H···*A*	*D*—H	H···*A*	*D*···*A*	*D*—H···*A*
O1—H1*O*1···O3^iii^	0.84	1.70	2.540 (3)	172
N1—H1*N*1···O2^iv^	0.91	1.90	2.785 (3)	163
N1—H2*N*1···O1^v^	0.91	2.37	3.110 (4)	138
N1—H2*N*1···O3^vi^	0.91	2.20	2.884 (3)	132
N1—H3*N*1···O3	0.91	1.84	2.732 (3)	164

Symmetry codes: (iii) −*y*+4/3, *x*−*y*+2/3, *z*−1/3; (iv) −*y*+4/3, −*x*+2/3, *z*+1/6; (v) *x*−1/3, *x*−*y*+1/3, *z*−1/6; (vi) −*x*+*y*+1/3, −*x*+2/3, *z*−1/3.
