# Supplementary figures and images for: Crystal structure of tris­(hydroxyl­ammonium) orthophosphate
Source: Acta Crystallogr E Crystallogr Commun. 2015 Oct 10;71(Pt 11):i10–1. doi: 10.1107/S2056989015018642 (PMC4645030; doi:10.1107/S2056989015018642)

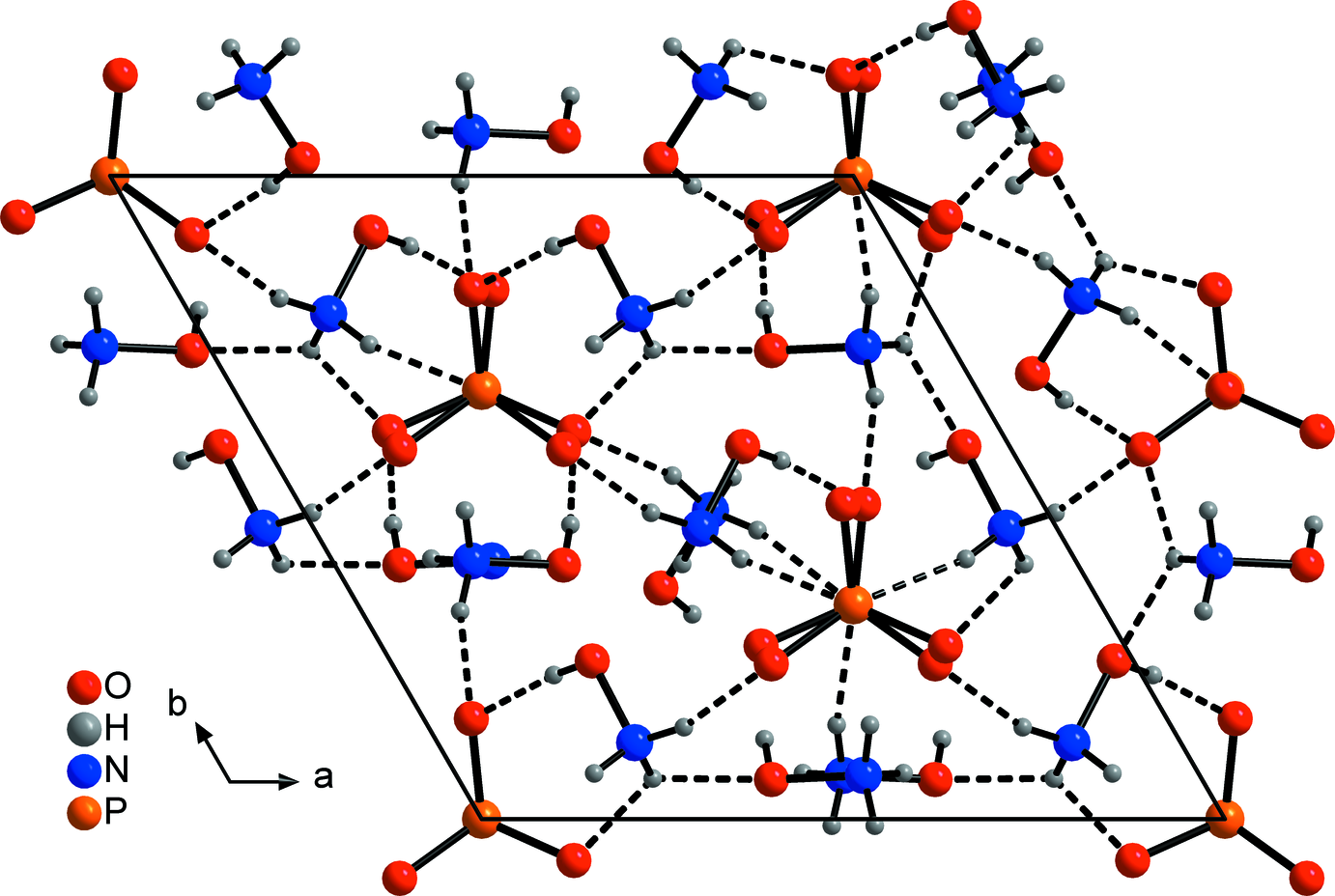

Supplement: Supplementary file 5 [file e-71-00i10-fig2.tif]
